# A New Long-Term Care Facilities Model in Nova Scotia, Canada: Protocol for a Mixed Methods Study of Care by Design

**DOI:** 10.2196/resprot.2915

**Published:** 2013-11-29

**Authors:** Emily Gard Marshall, Michelle Anne Boudreau, Jan L Jensen, Nancy Edgecombe, Barry Clarke, Frederick Burge, Greg Archibald, Anthony Taylor, Melissa K Andrew

**Affiliations:** ^1^Primary Care Research UnitDepartment of Family MedicineDalhousie UniversityHalifax, NSCanada; ^2^Primary Care Research UnitDepartment of Family MedicineDalhousie UniverstiyHalifax, NSCanada; ^3^Emergency Health ServicesDivision of Emergency MedicineDalhousie UniversityHalifax, NSCanada; ^4^Dalhousie University School of NursingSchool of NursingDalhousie UniversityHalifax, NSCanada; ^5^Integrated Continuing CareDepartment of Family MedicineDalhousie UniversityHalifax, NSCanada; ^6^Department of Family MedicineDalhousie UniversityHalifax, NSCanada; ^7^Oakwood Terrace Nursing HomeHalifax, NSCanada; ^8^Division of Geriatric MedicineDalhousie UniversityHalifax, NSCanada

**Keywords:** mixed methods, framework analysis, primary care, long-term care

## Abstract

**Background:**

Prior to the implementation of a new model of care in long-term care facilities in the Capital District Health Authority, Halifax, Nova Scotia, residents entering long-term care were responsible for finding their own family physician. As a result, care was provided by many family physicians responsible for a few residents leading to care coordination and continuity challenges. In 2009, Capital District Health Authority (CDHA) implemented a new model of long-term care called “Care by Design” which includes: a dedicated family physician per floor, 24/7 on-call physician coverage, implementation of a standardized geriatric assessment tool, and an interdisciplinary team approach to care. In addition, a new Emergency Health Services program was implemented shortly after, in which specially trained paramedics dedicated to long-term care responses are able to address urgent care needs. These changes were implemented to improve primary and emergency care for vulnerable residents. Here we describe a comprehensive mixed methods research study designed to assess the impact of these programs on care delivery and resident outcomes. The results of this research will be important to guide primary care policy for long-term care.

**Objective:**

We aim to evaluate the impact of introducing a new model of a dedicated primary care physician and team approach to long-term care facilities in the CDHA using a mixed methods approach. As a mixed methods study, the quantitative and qualitative data findings will inform each other. Quantitatively we will measure a number of indicators of care in CDHA long-term care facilities pre and post-implementation of the new model. In the qualitative phase of the study we will explore the experience under the new model from the perspectives of stakeholders including family doctors, nurses, administration and staff as well as residents and family members. The proposed mixed method study seeks to evaluate and make policy recommendations related to primary care in long-term care facilities with a focus on end-of-life care and dementia.

**Methods:**

This is a mixed methods study with concurrent quantitative and qualitative phases. In the quantitative phase, a retrospective time series study is being conducted. Planned analyses will measure indicators of clinical, system, and health outcomes across three time periods and assess the effect of Care by Design as a whole and its component parts. The qualitative methods explore the experiences of stakeholders (ie, physicians, nurses, paramedics, care assistants, administrators, residents, and family members) through focus groups and in depth individual interviews.

**Results:**

Data collection will be completed in fall 2013.

**Conclusions:**

This study will generate a considerable amount of outcome data with applications for care providers, health care systems, and applications for program evaluation and quality improvement. Using the mixed methods design, this study will provide important results for stakeholders, as well as other health systems considering similar programs. In addition, this study will advance methods used to research new multifaceted interdisciplinary health delivery models using multiple and varied data sources and contribute to the discussion on evidence based health policy and program development.

## Introduction

### Background

Until recently, people living in long term care facilities (LTCF) in the Capital District Health Authority (CDHA), Halifax, Nova Scotia, were responsible for finding their own family physician for primary care. Residents moving into LTCF could keep their existing family physician if the physician was willing and able to provide care in the LTCF. Otherwise, the resident had to find a local family physician who would agree to provide care prior to admission; often leading to admission delays. With many different family physicians providing care to a small number of residents in each facility, challenges arose with access to care, team communication, care planning, and coverage in emergency situations. Numerous studies have demonstrated that uncoordinated models of primary care in LTCF are less effective than those that are well-coordinated, which can result in limited access to proper primary care, and lead to suboptimal outcomes for elderly residents, particularly for end-of-life care [1-6].

In 2006, the Primary Care of the Elderly (PCOE) project was conducted to examine long-term care in CDHA. The PCOE project included a formal and grey literature review to identify potential models for providing primary care to the elderly, focus groups with nurse practitioners, family physicians, directors of care from continuing care facilities, staff of continuing care facilities, family members of frail elderly people, geriatricians, peer discussions, and a retrospective data review that were used to develop a new model of care called “Care by Design” [7]. Details of the PCOE project and findings can be found in their final report [7]. The project identified several concerns including: high rates of transfers from LTCF to emergency departments, even among those with “do not transfer” orders, lack of consultation with family physicians, and high rates of polypharmacy (the administration of multiple drugs at the same time for one or more health conditions) [7].

**Figure 1:**
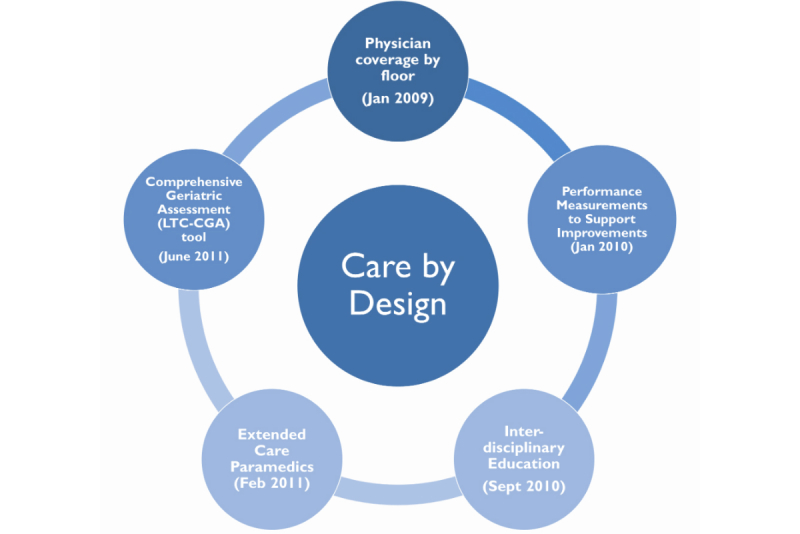
Care by Design elements and dates of implementation.

In January of 2009, the CDHA implemented a new model of care, known as Care by Design. This model included several important elements phased in over two years: (1) assigning all patients on one LTCF floor or wing the same physician and establishing a clear system of 24 hour on-call physician coverage; (2) designing measures to evaluate program performance; (3) implementing a program of standardized Comprehensive Geriatric Assessment for every resident using a tool designed specifically for the LTCF setting (the “LTC-CGA” – see Multimedia Appendix 1); and (4) an interdisciplinary team approach to the primary care (Multimedia Appendix 8). Specifically, interdisciplinary education has been an ongoing fluid part of Care by Design, directed by the co-leadership of the Long-Term Care Medical Advisory Committee and the District Council of Continuing Care on program specific elements such as clinical guidelines provided to each facility to be used along with education for staff on common conditions (eg, diabetes, hypertension, etc). In addition, an Extended Care Paramedic (ECP) program was introduced in collaboration with Emergency Health Services (EHS). The ECP program features a dedicated team of specially trained (or “extended care”) paramedics who respond to LTCF calls in order to address urgent care needs on-site to the greatest possible extent, and to aid in the coordination of planned transfers to hospital when necessary. Each of these elements was designed to address concerns identified in the original PCOE report (see Figure 1).

The quality improvement project, now called the Care by Design program, was initiated by front line family doctors with geriatric training. There was strong conviction that the change needed could be accomplished using some local models of care from the Veteran’s LTCF in Halifax, Nova Scotia, and an international model of care developed in the Netherlands nursing home medicine programs [8]. Funds were requested from CDHA for the PCOE project to define the problems and plan the intervention. The PCOE report was presented to the Nova Scotia Department of Health and Wellness, which provided funding for the first few years and since the program was successful, CDHA continued to support the program. The impetus for this quality work for both the Department of Health and Wellness and CDHA was mostly the high transfer rates of residents to emergency departments and associated high costs without clear clinical or system gains.

This paper outlines the protocol for a comprehensive inter-sectorial study of the Care by Design and ECP model, using a concurrent triangulation mixed methods approach. Care by Design is a coordinated model of primary and urgent care in LTCF that is unique in Canada. Its implementation provided an ideal opportunity to study this initiative by measuring patient and system outcomes with a quantitative time series design, along with a concurrent qualitative exploration of the experiences of multiple stakeholder groups. Data collection is to be completed by early fall 2013.

### Objectives

This study has three main objectives: (1) to measure the effect of the new Care by Design model in LTCF and its major components; (2) to understand how key stakeholders experience the new Care by Design model components; and (3) to illuminate how the structure and process influence the outcomes for residents and care providers.

## Methods

### Mixed Methods Design

**Figure 2:**
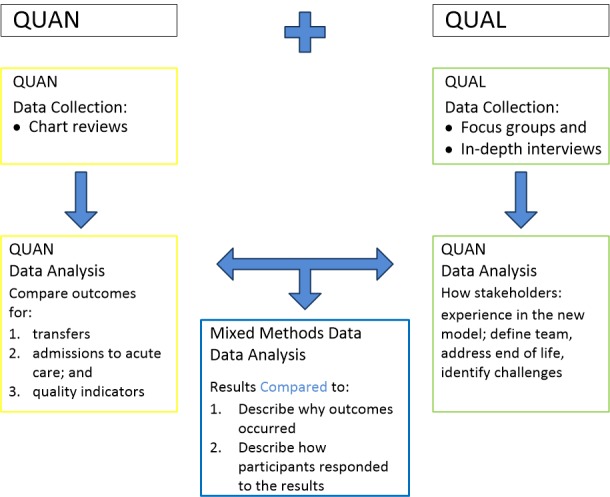
Concurrent Triangulation Design (adapted from Creswell and Clark 2011).

The mixed methods design employs a concurrent triangulation model [9] giving equal priority to our concurrently collected qualitative (QUAL) and quantitative (QUAN) data (see Figure 2).

As per the conventions of mixed method notation, QUAN refers to quantitative methods and QUAL refers to qualitative methods (Figure 2). The capitalization refers to each method being a significant contributor, rather than QUAN-qual where the quantitative methods would take precedence.

This study employed a mixed methods approach which included focus groups, in-depth semi-structured interviews, and an observational time series study. The mixed methods approach lends itself to a more comprehensive inquiry [10] to answer questions that one method alone cannot address. For example, while the quantitative data collection obtains the numbers of events that took place (ie, number of ambulance transports to emergency departments, number of visits with family physicians, number of medications prescribed, etc), it is the qualitative methods that delves into the complexity of health, health care, and the environment in which these events took place, helping to answer questions about why events occurred and what relationships exist between events. For the study to be truly mixed methods, the two approaches must be integrated [11-13].

Mixed methods is a valuable approach for this study, as the data gathered during the qualitative phase of the study informs the findings of the quantitative chart review. Similarly, the quantitative results demonstrate the outcomes related to perceptions and experiences shared by participants in the findings of the qualitative data.

### Setting

In 2011, the Halifax, Nova Scotia census metropolitan area had a population of 390,328 people, with 13.1% aged 65 or older [14]. It is predicted that by 2036, 30% will be 65 and older, with 10% being over 80 years of age [15]. The CDHA is responsible for delivering core health services in the Halifax Regional Municipality. Ten of the twelve Care by Design participating LTCF located in the CDHA participated in the study. Two CDHA LTCF were excluded because their model of primary care differed from Care by Design in important ways which would make them difficult to compare: one is a teaching facility and one has a full-time “nursing home physician”.

### Quantitative Approach

#### Overview

The retrospective chart abstraction data are being collected from three time periods: time period 1: September 1, 2008-February 28, 2009 (before Care by Design or ECP programs in place); time period 2: September 1, 2010-January 31, 2011 (after Care by Design program implemented, before ECP program started. The original intent was to collect comparable six months of data from September 1, 2010 to February 28, 2011; however, due to early ECP training there was too much overlap in services for the month of February 2011); and time period 3: September 1, 2011-February 29, 2012 (after both Care by Design and ECP programs started; see Multimedia Appendix 2).

These time periods were chosen to reflect the implementation of different components of the new model of care, and to include the same months in each year in order to minimize confounding by seasonality (eg, transfer rates were expected to climb during influenza season each year). Data will be obtained using chart abstraction from three sources: LTCF charts, EHS records, and hospital emergency department and acute care charts.

#### Chart Abstraction Tool Development

The starting point of our chart abstraction tool development is the PCOE project that examined the issue of LTCF to emergency department transfer in CDHA. As part of the PCOE project, a literature review was conducted and a paper chart abstraction tool with numerous indicators was developed and used. Our study team reviewed the PCOE tools, and revised them based on our newly developed objectives and research questions (see Table 1). The revised indicators were included in an Access database created for ease of data entry (Multimedia Appendix 9).

**Table 1:** Research questions.

Research questions		
**Quantitative: What changes in health care outcomes are observed pre/post implementation of the new Care by Design model in LTCF?**		
	Is there a reduction in ambulance transfers to hospital with the new model of care?	
	Is there a reduction in the transfer of “comfort care” residents (for whom transfer to acute care is explicitly not part of the established goals of care) to hospital with the new model?	
	Is there a reduction in the rates of polypharmacy?	
	Is there a reduction in falls with the new model of care?	
	Is there improvement in wound care protocol adherence with new model of care?	
	Do we see increased care team communication recorded in charts?	
	Do we see a reduction in the number of attempts to contact family physicians to attend critical incidents with the new model?	
**Qualitative: How do family doctors, nurse practitioners, registered nurses (RN), licensed practical nurses (LPN), continuing care assistants/personal care workers (CCA/PCW), administration and staff, ECP, and residents/families experience the new Care by Design model?**		
	What challenges exist under the new model?	
	How do the various stakeholders define the team? What would their ideal care team comprise and how would it function?	
	How does the model affect end-of-life care? (ie, Do families know who to talk to about end-of-life questions and planning? Are “comfort care” requests known and followed?)	
**Knowledge Translation: How well-implemented and useful is the LTC-CGA as a knowledge translation tool?**		
	**Process goal. To study the implementation of the LTC-CGA tool as follows:**	
		What is the experience and perceived value of educating primary care physicians and nurses about the importance of the tool and how to use it? (eg, was training experienced as sufficient and well-implemented?)
		What is the uptake of the LTC-CGA (ie, completion rates and completeness of all sections)?
		Is the LTC-CGA acceptable to users?
		Is the new billing code for LTC-CGA completion being used?
	**Outcome goals:**	
		To test the efficacy of the LTC-CGA (ie, does its use improve care for older adults who live in LTCF)? Specific elements to be studied include usefulness in defining goals of care and impact on clinical care (eg, whether it accompanies residents transferred to emergency department, hospital admissions and inter-facility transfers).
		Is the LTC-CGA useful for end-of-life discussions and planning?
**Mixed Methods: How do structure and process influence outcomes for residents and care providers?**		
	Which aspects of the new model are perceived to be attributed to changes observed in the chart review data by different stakeholders?	
	Do providers, administrators, and residents feel a reduction in ambulance transfers to emergency department (if found)? Is it indicative of better care for residents? Under what parameters would a reduction in transfers been experienced as improved care? What issues remain associated with ambulance transfers to emergency department under the new model of care in LTCF? (ie, access to physicians, communication, meeting the wishes and needs of residents, services provided by paramedics).	
	How do the various stakeholders experience the projected increase of residents dying in place in the LTCF?	
	What do stakeholders say about the ease-of-use and helpfulness of the LTC-CGA tool for team communication, care planning, and communication between providers and residents/family members? How does the completeness of the LTC-CGA reflect and have an impact on the experiences of stakeholders?	
	How is care team communication found in the chart reviews experienced by stakeholders? Are the experiences of team care approach under the new model captured in the chart review data?	

#### Outcomes and Data Elements

Outcomes addressing specific research questions are categorized into three key areas: (1) system outcomes; (2) quality of clinical care; and (3) safety outcomes (see Table 2). Primary outcomes include transports of LTCF residents to emergency departments, polypharmacy (prevalence will be defined as 6 or more medications, a widely accepted definition that is associated with increased risk of inappropriate medication use and medication-related harms) [16], fall rates, wound care protocols, team communication, and provider contact.

**Table 2:** Key outcome measures and data source.

Category	Outcome measure	Data source
System Outcomes	Reason for 911 call (ie, breathing, falls, other)	LTCF charts
	Percentage of patients transported who had no visit from a family physician in LTCF within 1 and 4 weeks prior to transport to emergency department	LTCF charts
	Number of times family physician attended a team meeting during study time period	LTCF charts
	Family physician visits to patient 3 months prior to most recent Emergency Health Service call	LTCF charts
	Number of notes in chart from family physician during time period	LTCF charts
	Health care profession who made on-site assessment	LTCF charts
	Any investigations (ie, diagnostic imaging, blood work, other) 7 days prior to Emergency Health Service call	LTCF charts
	LTC-CGA present	LTCF charts; Hospital charts
	Percentage of cases where facility was able to reach the family physician prior to Emergency Health Service call	LTCF charts
	Percentage of cases with an onsite assessment by a family physician prior to Emergency Health Service call	LTCF charts
	Number of times Emergency Health Service (ECP and/or emergency paramedics) involved during time period	LTCF charts
	Number of patients transported to emergency department by ambulance	LTCF charts; EHS database
	Proportion of patients who are transported to emergency department who have advance comfort care directive requesting no transfer to hospital/acute care	LTCF charts; EHS database
	Whether LTC-CGA sent with resident to emergency department	Hospital charts
	If ECP involved in call	EHS database
	If ECP involved, whether they consulted with EHS physician	EHS database
	If ECP involved, whether they consulted with family physician	EHS database
	Length of Emergency Health Service call	EHS database
	Ambulance offload time in emergency department	EHS database; Hospital charts
	Length of stay in emergency department	Hospital charts
	Percentage of residents who were transported to emergency department that were admitted to hospital	Hospital charts
	Length of stay in hospital	Hospital charts
	Percentage of transferred residents who returned to LTCF upon hospital discharge	LTCF charts; Hospital charts
Clinical and Quality of Care	Number of assessments and treatments provided by Emergency Health Service	EHS database
	Admitting diagnosis	Hospital charts
	Death rate in hospital	Hospital charts
	Influenza vaccination rates	LTCF charts
	Rates of falls	LTCF charts
	Pressure wound care	LTCF charts
	Polypharmacy rates	LTCF charts
Safety Outcomes	Relapse rate back to Emergency Health Service system (number of patients seen by ECP and/or paramedics and not transported who had unexpected repeat 911 call made for them within 48 hours for a related reason)	EHS database

#### Participants

We will proportionally sample and review a minimum 200 of the possible 1482 charts from LTCF (approximately 13% of all charts) during time periods 1 and 3 for whom a call to 911 for ambulance service was not made. We will also review all charts from hospital/ED transfers for all three time periods (estimated to be approximately 250 per time period). In addition, records of all hospital transfers during the study period will be reviewed from both emergency department and acute care charts.

#### Quantitative Data Collection

Retrospective chart reviews will be conducted by three trained nurse research assistants on all residents’ charts that had a 911 call from participating LTCF during the three time periods. In addition, a comparison stratified sample of 100 control LTCF charts of residents who did not have a 911 call made for them will be abstracted from time periods 1 and 3. These control charts are included to determine if any observed changes in access and coordination of care are the same as those who had 911 calls and will be stratified to be proportional to the size of the facility.

Data are being abstracted from three sources: LTCF charts, acute care charts, and an EHS database. Deterministic data linkage is used to match records from the three sources [17]. The data query begins with those with an emergency department transfer from an EHS database, which included records for all residents at participating LTCF who had a 911 call. The following data elements are provided for the purpose of linking EHS data to acute care and LTCF data: Emergency Health Service call identifier (to link to complete EHS dataset), health card number, date of service, date of birth, and location of service. Capital District Health Authority medical records required health card number, date of service, and date of birth to identify patient charts. Location of service is being used to ensure that the call was indeed from a LTCF. The health card number is the unique identifier to link datasets. If this element is missing from any of the data sources, the research associate will judge records as matching if date of service, date of birth, and location of service matched. Control charts of resident for whom 911 was not called will be reviewed as follows: 100 charts for time period 1 and 100 charts for time period 3, stratified proportionally from the difference LTCF based on their proportional number of beds.

A random sample of 10% of charts will be re-abstracted to assess inter-rater reliability between chart abstractors. LTCF charts had both paper and electronic format, depending on the LTCF and time period. All acute care charts existed within an electronic charting system. All chart abstraction data will be entered into a Microsoft Access database on a password-protected laptop computer. Data will then be entered into SPSS 20, cleaned, and prepared for analysis.

#### Planned Quantitative Analysis

Descriptive statistics for outcome measures by time period will be explored initially for each outcome. Comparison of proportions and rates between time periods will be conducted. For example, chi-square analysis will be used to see if there is a change in the proportion of LTCF transports to emergency departments between time 1 and time 2. In another analysis, “number of physician notes in chart” will be turned into a categorical variable, and rates will be compared between time periods using chi-square and associated tests of strength of association (eg, eta). Later, we will explore multivariate regression models to predict primary outcomes. Types of regression models, such as continuous, logistic, and hierarchical modeling will be determined by the covariates chosen, and in relation to the research question under examination. For example, to predict LTCF transports to emergency departments, a logistic regression model would be developed using appropriate independent variables such as number of physician visits, frailty scores and polypharmacy. Significance is set a priori at *P*<.05.

### Qualitative Approach

#### Overview

Qualitative data are collected through focus groups and individual interviews. Focus group and interview schedules were developed in consultation with the full research team. Digital audio recordings are transcribed verbatim and entered into Atlas.ti software for analysis.

#### Participants-Qualitative

Focus group and interview participants live, work, or have a loved one living in a LTCF. They include a variety of key stakeholders – family physicians, nurse practitioners, RNs, LPNs, CCA/PCWs, residents, family members, ECP, and LTCF administrators. In the first stage, 11 focus groups were held with a range of 3-10 participants in each focus group, for a total of 75 key stakeholder focus group participants. One focus group was held for each of Care by Design physicians, LTCF administrators, and ECP. A total of 3 focus groups were conducted with RNs and LPNs, 2 with CCA/PCWs, and 3 with residents and/or family members. With an interview schedule based on preliminary analysis of focus group data, a total of 40 key stakeholders are participating in in-depth interviews (10 residents and/or family members; 3 administrators; 18 nurses (RNs & LPNs); 8 CCA/PCWs; 1 nurse practitioner, and 1 physician decision maker).

#### Qualitative Data Collection

In the qualitative phase of the study we explore the lived experience under the new model from the perspectives of key stakeholders including family physicians, RNs, LPNs, LTCF administrators, CCA/PCWs, and ECP, as well as residents and family members (see focus group guides Multimedia Appendices 3-7). The qualitative component sheds light on how the model is working to meet the needs of providers and residents and where challenges still remain. This includes: (1) an exploration of how care teams are defined and experienced from the varied stakeholder perspectives; (2) whether the LTC-CGA facilitates better care for LTCF residents; and (3) how the new model functions in terms of end-of-life planning and communication between team members and with residents and families. The qualitative work considers recommendations for improvement and highlights significant benefits of the new model from the perspectives of key stakeholders.

#### Qualitative Analysis

Transcribed qualitative data is subject to rigorous data quality checks [18]. Transcriptions are entered into Atlas.ti qualitative data analysis software [19]. Data will be coded using an agreed-upon coding scheme developed by the research team. Thematic analysis, which allows for thematic coding within a structured framework approach, using open and axial coding to identify themes and categories as they emerge from the data will be used. Framework analysis [11, 20] will be conducted of narrative responses to open-ended questions about primary care and outcomes in LTCF, the acceptability of the Care by Design and ECP model, and its impact on clinical care.

#### Interpretive Lens: Framework Analysis

A framework analysis approach, as described by Ritchie and Spencer (1994), will be used to describe and detect the phenomenon of interest [21]. This is a qualitative method of data analysis that is well-suited to research that has specific research questions, limited time frame, specific sample, and predefined issues [22]. It is particularly useful for explanatory analysis and understanding individual outcomes within a systems perspective [23]. Continually revisiting the question “what are the participants trying to describe?” allows the researcher to code for themes and describe the phenomenon in participants’ own words. Use of this method allows the researcher to organize data while maintaining the original context and observations in which the data occurs, and to facilitate a systematic analysis [23], making it possible to track the researchers’ interpretations, thus maintaining transparency and enhancing validity of the findings [24].

Framework analysis involves a series of five connected but distinct stages [21,22,25]. Data are sorted, charted, and sifted in accordance with key issues and themes, relying on the intuition and creativity of the analyst to see linkages among them. There is sufficient flexibility that analysis can wait until all data collection is complete, or it can begin part way through the study. Familiarization (step 1) will begin with becoming very familiar and comfortable with the data. These will be in the form of transcripts, written observations, and field notes. This will allow the analyst to become aware of any recurrent themes in the data. Development of a thematic framework (step 2) would follow. Emerging themes from within the data set form the basis of the thematic framework and can be used to sort and manage the data. Early consideration of connections between responses and relevance of identified issues begins at this stage. Careful notes of the framework development will be taken to increase transparency. The framework will be applied systematically, incorporating new data as it emerges (step 3). Step 3 is part of indexing, a process by which individual pieces of data are applied to the framework developed in step 2, allowing for easy access to the original context in which it was discovered. Charting (step 4) allows the researcher to build an understanding of the data as a whole. Data segments or quotes are arranged systematically in a chart or matrix according to the appropriate theme. Charts will include headings and subheadings developed in the thematic framework. The final stage (step 5), mapping and interpretation, involves analysis of key characteristics of the data, including associations between themes and creation of typologies.

The theoretical approach will incorporate Donabedian’s classic structure, process, outcome framework for assessment of quality of care as it is widely applied in health services research field [26-28]. This framework, informed by Donabedian’s framework, will assist with analysis and interpretation to ensure aspects of structure, process, and outcomes are assessed and linked [26-28].

### Planned and Potential Applications of Study Data

This work will have a wide range of applications for care providers, health care policy decision-makers, and research. It will provide analysis of the changes in LTCF health services and the impact on the healthcare, experience of stakeholders, quality of care, and possibly LTCF residents’ quality of life. In addition, it will provide baseline data for improved health care planning in LTCF in Nova Scotia.

*Applications for care providers*: this study examines the health care and health outcomes of interdisciplinary care professionals from RNs, LPNs, CCA/PCWs, family physicians, and paramedics in LTCF. The results will inform aspects of the best practice related to primary care and team role integration. Results from this study not only provide insight into the impact of the changes resulting from Care by Design, but also identify areas for expansion and refinement of the model.

*Application for health care system*: this study examines the utilization of health care services and health professionals by residents in LTCF. Findings from this study will enable informed health care planning decisions and stimulate reform of legislation and regulation governing long-term health care delivery within the province.

*Applications for program evaluation of quality improvement*: as we plan for the health care and residential needs of our aging population, studies of this nature will be integral in developing methodology and findings related to program evaluation and quality improvement. This will be illustrated by analysis of the quantitative data at set intervals when new components to CBD were added. The data will provide information on the impact of each change on health services delivery in LTCF. The qualitative data will provide insight as to the nature of that impact and may identify areas for further quality improvement.

*Applications for research*: this work is an example of successful mixed methods evaluation research incorporating qualitative, quantitative, and mixed methods research questions and analytic techniques which may be useful to future research examining complex models of care. It provides a method of evaluation to compare other models of care for LTCF to further develop best practices. This method of evaluation will allow ongoing evaluation of future changes in the CBD model to maximize resource utilization in a cost effective manner.

In today’s health care settings, there is a need to provide high-level, efficient, and quality care at all levels of a rapidly changing health care sector. This project will provide an example of evaluation of complex systems. They can be evaluated as change is occurring, providing comprehensive information of both quantitative and qualitative aspects of the change.

## Discussion

### Summary

Using mixed methods design, this study of Care by Design will add to the literature using methods to evaluate new models of primary care, and will contribute to the discussion on evidence-based health policy development, and will provide direct feedback to Care by Design stakeholders. In the mixed methods design different sources of time series chart abstraction data (over three time periods) relating to the implementation of Care by Design, and qualitative data from focus groups and interviews are combined for a comprehensive analysis. Led by an interdisciplinary research team, this multifaceted study design allows for examination of long-term care at several levels including system and structural elements, process elements including team collaboration and coordination, role integration, and care plan development and implementation; as well as analysis of health care and health status outcomes across LTCF residents. This study provides insight and recommendations at all levels of long-term care delivery and assesses the success and challenges of the components of Care by Design and ECP.

### Research Team

The interdisciplinary research team consists of experienced primary care researchers, a geriatrician-researcher, health services researchers, and care providers (family physicians, paramedics, emergency physicians, nurse practitioners, and nurses), long-term care administrators and residents’ advocate. There is a strong representation of methodological skills including mixed methods, qualitative methods (including both focus groups and interviews), statistical analysis, chart abstraction, and large database analysis experience.

## Multimedia Appendix 1

Long-term care comprehensive geriatric assessment Tool.

## Multimedia Appendix 2

Care by Design study periods.

## Multimedia Appendix 3

Focus group guide – administration and administrative staff.

## Multimedia Appendix 4

Focus group guide – nurses.

## Multimedia Appendix 5

Focus group guide – extended care paramedics.

## Multimedia Appendix 6

Focus group guide – physicians.

## Multimedia Appendix 7

Focus group guide – residents and/or family members.

## Multimedia Appendix 8

Care by Design path.

## Multimedia Appendix 9

Key Indicators.

## Abbreviations

CCA: continuing care assistant
CDHA: Capital District Health Authority
ECP: extended care paramedics
LPN: licensed practical nurse
LTCF: long-term care facility
LTC-CGA: Long-Term Care Comprehensive Geriatric Assessment
PCOE: Primary Care of the Elderly
QUAL: qualitative
QUAN: quantitative
