# Supplementary material for: A New Long-Term Care Facilities Model in Nova Scotia, Canada: Protocol for a Mixed Methods Study of Care by Design
Source: JMIR Res Protoc. 2013 Nov 29;2(2):e56. doi: 10.2196/resprot.2915 (PMC3869043; doi:10.2196/resprot.2915)
Supplement: Supplementary file 1 [file resprot_v2i2e56_app1.pdf]

Appendix A – Long-term Care Comprehensive Geriatric Assessment Tool

Comprehensive Geriatric Assessment (CGA) is a validated method for assessing frail older adults in ambulatory and acute care environments which embraces this complexity [29, 30, 31]. The CGA is a holistic assessment which documents an older patient's health status, including cognition (e.g., dementia, delirium), mood, mobility, function, appetite, weight, bowel and bladder function, medical conditions, and medications [32].

A version of the CGA, the "LTC-CGA" (Long-Term Care Comprehensive Geriatric Assessment), has been modified and validated for use in LTCF [33]. Modifications from the original CGA to better suit the LTCF setting include documentation of behavioural disturbances common in dementia, foot and dental care requirements, skin integrity, if a legal next of kin has been appointed, and goals of care (e.g., whether resuscitation is to be attempted or hospital transfer for acute illness). The LTC-CGA currently exists in a one page paper format on the resident's chart (see figure 4).

As part of a larger "Care by Design" program, the LTC-CGA was implemented in LTCF within Capital District Health Authority in Nova Scotia, with the aim of improving quality of care and health outcomes for frail older residents of LTCF. Family physicians caring for LTCF residents are mandated to complete the LTC-CGA for every resident, and to keep it updated every six months and after any significant change in health status. The tool is meant to have a prominent place in the LTCF chart in order to guide care in that setting, and it is also designed to accompany residents who are transferred between facilities and to acute care in order to ensure clear communication of baseline health and function. The LTC-CGA has the potential to be a powerful tool for assessment and communication in LTCF, however its uptake (i.e., is it completed fully, is it accompanying residents who are transferred to acute care settings?) and acceptability (i.e., is it easy to use? Useful? Meeting needs?) to end users are not known.

Capital Health

Long-Term Care Comprehensive Geriatric Assessment Form

WNL = Within Normal Limits   ASST = Assisted
IND = Independent   DEP = Dependent

Cr Cl: ____________

Infection Control
☐ MRSA
☐ VRE
☐ Flu shot given
☐ Pneumococcal vaccine given
☐ TB test done
☐ Tetanus/Diphteria

Chief lifelong occupation:____________   Education: (years)______

Cognitive Status
☐ WNL
☐ Dementia
☐ Other
MMSE ________

Emotional
☐ WNL   ☐ ↓Mood
☐ Depression   ☐ Anxiety
☐ Other   ☐ Hall/Del

Behaviours
☐ Verbal Non-aggressive
☐ Verbal Aggressive
☐ Physical Non-aggressive
☐ Physical Aggressive

Communication:

Speech
☐ WNL
☐ Impaired

Hearing
☐ WNL
☐ Impaired

Vision
☐ WNL
☐ Impaired

Strength
☐ WNL   ☐ Weak   Upper: PROXIMAL DISTAL R L
Lower: PROXIMAL DISTAL R L

Footcare needed
☐ Y ☐ N

Dental care needed
☐ Y ☐ N

Skin Integrity Issues
☐ Y ☐ N

Legal NoK:______________

Advanced Directives ☐ Y ☐ N

Code Status
☐ Do Not Attempt to Resuscitate
☐ Do Not Hospitalize
☐ Resuscitate

**Mobility**	Transfers	IND	ASST	Dep
	Walking	IND Slow	ASST	Dep
	Aid	________		
**Balance**	Balance	WNL	Impaired	
	Falls	N Y	Frequency	
**Elimination**	Bowel	CONSTIP	CONT	INCONT
	Bladder	CATHETER	CONT	INCONT
**Nutrition**	Weight	STABLE	LOSS	GAIN
	Appetite	WNL	FAIR	POOR
**ADLs**	Feeding	IND	ASST	Dep
	Bathing	IND	ASST	Dep
	Dressing	IND	ASST	Dep
	Toileting	IND	ASST	Dep

Marital Status
☐ Married
☐ Divorced
☐ Widowed
☐ Single

Family Stress
☐ None
☐ Low
☐ Moderate
☐ High

Problems/Past history	Med adjust req.	Associated Medication
1. ____________	○	____________
2. ____________	○	____________
3. ____________	○	____________
4. ____________	○	____________
5. ____________	○	____________
6. ____________	○	____________
7. ____________	○	____________
8. ____________	○	____________
9. ____________	○	____________
10. ____________	○	____________
11. ____________	○	____________
12. ____________	○	____________
13. ____________	○	____________
14. ____________	○	____________
15. ____________	○	____________

Current Frailty Score

Scale
1. Mildly frail ☐ 2. Moderately frail ☐ 3. Severely frail ☐ 4. Very severely ill ☐ 5. Terminally ill ☐

Physician: ________________   Date: ________ YYYYMMDD

CD0184MR_06_

© 2007-2008 All rights reserved, Geriatric Medicine Research, Dalhousie University.Halifax, Canada.
Used with permission

Figure 4 Long-Term Care Comprehensive Geriatric Assessment
