# Supplementary figures and images for: A New Long-Term Care Facilities Model in Nova Scotia, Canada: Protocol for a Mixed Methods Study of Care by Design
Source: JMIR Res Protoc. 2013 Nov 29;2(2):e56. doi: 10.2196/resprot.2915 (PMC3869043; doi:10.2196/resprot.2915)

## Appendix B: Care by Design Time Periods

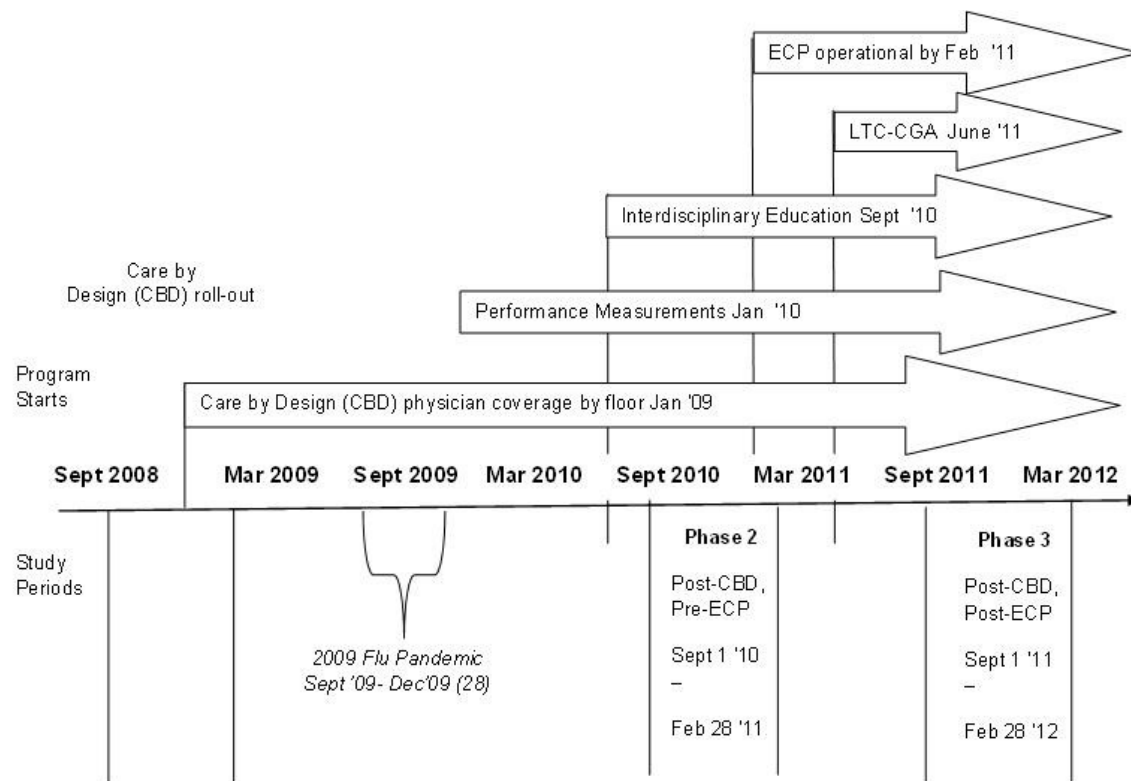

Supplement: Supplementary file 2 [file resprot_v2i2e56_app2.pdf]

Figure 1: Care by Design elements and dates of implementation

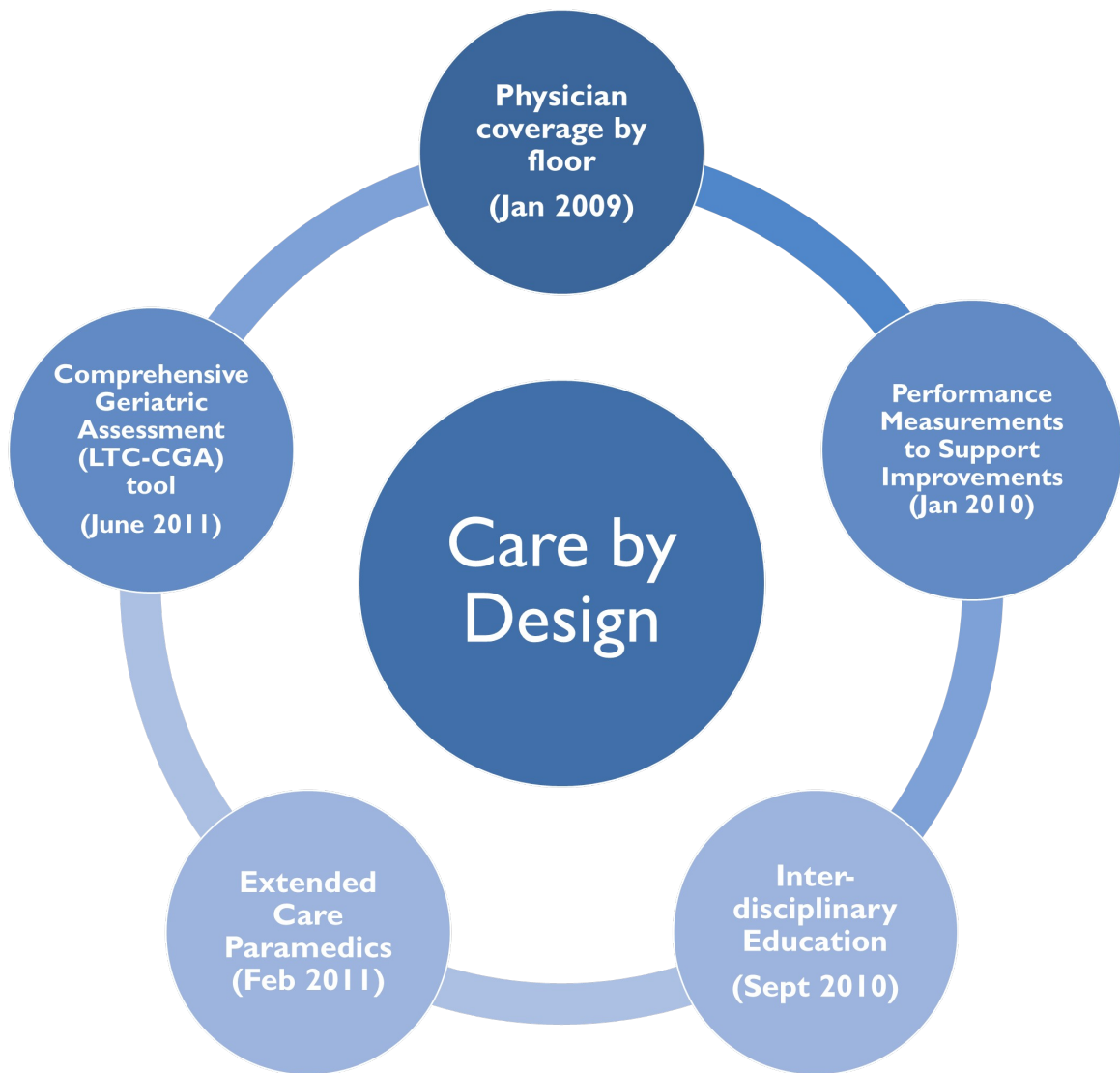

Supplement: Supplementary file 8 [file resprot_v2i2e56_app8.pdf]
