# Supplementary material for: A New Long-Term Care Facilities Model in Nova Scotia, Canada: Protocol for a Mixed Methods Study of Care by Design
Source: JMIR Res Protoc. 2013 Nov 29;2(2):e56. doi: 10.2196/resprot.2915 (PMC3869043; doi:10.2196/resprot.2915)
Supplement: Supplementary file 3 [file resprot_v2i2e56_app3.pdf]

Appendix C – Focus Group Guide – Administration and Administrative Staff

Archival #

Site: Moderator:

Number of Participants: Note-Taker:

Date: Transcriber:

Start Time: End Time:

First I'd like to thank you all for coming here to speak with us today about your experiences with the new model of care, Care by Design, being implemented in long term care facilities in the Capital District Health Authority. The Care by Design model involves having a dedicated primary care physician assigned to each floor of a long-term care facility and uses a team approach to care. My name is Michelle, and we are here from Dalhousie University to talk about your experiences with this new model of care. We want to know how the model is being experienced by and how it is affecting those who work in long-term care facilities, the residents, and their family members.

We will be asking you a series of questions. Questions will be posed to the group and it will be your choice if you'd like to answer. At any time, please feel free to pass on a question you don't feel comfortable answering. The session should take no more than 2 hours and we can take a 10-minute break in the middle if the group would like one. There are a lot of people here today so we need to ensure that everyone gets a chance to speak. We would like to ask you to speak one at a time and to refrain from interrupting. If more than one person wants to answer a question, please raise your hand and we will establish an order. Please be respectful of each other and acknowledge the right for people to express different opinions. Everyone may have a different opinion or answer to the question we ask; I would like to hear all of these opinions! Everything we say in this focus group is considered confidential, and we will not be identifying any individuals when reporting our findings. Since you are here today in a group it is important that each of you respect others' right to privacy and not discuss who took place in the focus group outside this room or what participants said during this focus group.

The session will be audio-taped. Only the researchers will have access to the recording. We have informed consent documents we require to have you read and sign.

Our role here today is to learn from you so please be open in sharing your experiences. Does anyone have any questions before we begin?

1. What is your experience of the service provided by the new model of care?

a) How has the model of care changed primary care provision at the facility?

b) What have been the challenges?
   Probe: Are there any services that are lacking that you would like to see included?
   Probe: Are there ways that the model could be changed to better meet your needs or the needs of the residents or family members?
   Probe: Are there any new challenges that came along with the introduction of this model? How can these challenges be overcome?

c) What is your vision for primary care in your facility?

2. What is your experience of primary care as provided by the model of care?

3. Can you tell us about the team that is involved in the care provided at your facility?

a) Who are the team members?

b) What are the roles of the team members?

c) Who would you ideally like to see as part of the team?

d) Can you tell us about your experiences with the Extended Care Paramedics?

e) Have you noticed changes with the team?
   Probe: Any differences in team creation and construction? Any differences in training as a team?

4. What is your experience of end of life care at your facility with the new model of care?

a) How do you know if someone is at end of life? What does that look like? What do you do?

b) How is end of life care coordinated?

c) How are families involved in end of life planning?

d) What is your experience of the sharing of knowledge about and following of comfort care requests?

5. How does communication with the care teams work in your facilities?

a) Tell us about the level of accessibility you have to physicians.
   Probe: When you have needed to speak to a doctor, how accessible was the doctor? Any suggestions for improvement?

b) What about nurses?

c) Describe how having a physician on site has affected the way the team communicates.

d) Describe how the LTC-CGA has affected team communication.

6. Can you tell us about any structural, institutional, or legislative factors that you find help you create the service you want or do you find them challenging?
