# Supplementary material for: A New Long-Term Care Facilities Model in Nova Scotia, Canada: Protocol for a Mixed Methods Study of Care by Design
Source: JMIR Res Protoc. 2013 Nov 29;2(2):e56. doi: 10.2196/resprot.2915 (PMC3869043; doi:10.2196/resprot.2915)
Supplement: Supplementary file 7 [file resprot_v2i2e56_app7.pdf]

Appendix G – Focus Group Guide – Residents and/or Family Members

Archival #

Site: Moderator:

Number of Participants: Note-Taker:

Date: Transcriber:

Start Time: End Time:

First I'd like to thank you all for coming here to speak with us today about your experiences with the new model of care, Care by Design, that was implemented in long term care facilities in the Capital District Health Authority. The Care by Design model involves having a dedicated primary care physician assigned to each floor of a long-term care facility and uses a team approach to care. My name is Michelle, and we are here from Dalhousie University to talk about your experiences with this new model of care. We want to know how the model is being experienced by and how it is affecting those who work in long-term care facilities, the residents, and their family members.

We will be asking you a series of questions. Questions will be posed to the group and it will be your choice if you'd like to answer. At any time, please feel free to pass on a question you don't feel comfortable answering. The session should take no more than 2 hours and we can take a 10-minute break in the middle if the group would like one. There are a lot of people here today so we need to ensure that everyone gets a chance to speak. We would like to ask you to speak one at a time and to refrain from interrupting. If more than one person wants to answer a question, please raise your hand and we will establish an order. Please be respectful of each other and acknowledge the right for people to express different opinions. Everyone may have a different opinion or answer to the question we ask; I would like to hear all of these opinions! Everything we say in this focus group is considered confidential, and we will not be identifying any individuals when reporting our findings. Since you are here today in a group it is important that each of you respect others' right to privacy and not discuss who took place in the focus group outside this room or what participants said during this focus group.

The session will be audio-taped. Only the researchers will have access to the recording. We have informed consent documents we require to have you read and sign.

Our role here today is to learn from you so please be open in sharing your experiences. Does anyone have any questions before we begin?

1. Can you tell us about your experiences with care in this facility?

a) Can you tell us about your experiences transferring into this facility?
   Probe: Did you have to change family physicians?
   Probe: If so, what was it like changing family physicians?

b) What was your experience like with admissions?

c) Did any of you have experience with long-term care before the new physician per floor model of care? If so, how is this experience different from what was in place before?

d) What is working well in the new way of doing things?

e) What could be improved?

Probe: Are there any services that are lacking that you would like to see included?

Probe: Are there ways that the new way of doing things could be changed to better meet your needs?

f) Could you share with us an experience you have had with the new service of care that affected your experience in long-term care?

2. Please tell us about your interactions with the health professionals in this facility:

a) The doctor

b) Nurses

i. Registered

ii. LPN

c) Continuing Care Assistants

d) Dietary

e) Extended Care Paramedics

f) Administration

g) Other

h) What is your experience when you need to talk to someone about care – the doctor, nurse, administration?

Probe: When you have needed to speak to a doctor or nurse, how accessible was the doctor or nurse? Any suggestions for improvement?

i) Are you familiar with the long-term care geriatric assessment (LTC-CGA) forms?

3. We recognize this next section on end of life care might not yet be applicable for all of your circumstances. Can you tell us about your experience of planning and making decisions about end of life care in relation to family members/self in this facility?

   a) Do you or family members know who to talk to about end of life questions and planning?

   b) Did the people you talked to share what to expect? Did they share options? Did you feel they were comfortable with end of life care?

   c) Are comfort care requests known and followed?

Is there anything we haven't covered yet today that you would like to talk about?
