# Supplementary material for: A New Long-Term Care Facilities Model in Nova Scotia, Canada: Protocol for a Mixed Methods Study of Care by Design
Source: JMIR Res Protoc. 2013 Nov 29;2(2):e56. doi: 10.2196/resprot.2915 (PMC3869043; doi:10.2196/resprot.2915)
Supplement: Supplementary file 9 [file resprot_v2i2e56_app9.pdf]

Table 2. Key Indicators

Category	Outcome Measure	Data Source
	Reason for 911 call (i.e., breathing, falls, other)	LTCF charts
System Outcomes	Percentage patients transported who had no visit from a family physician in LTCF within 1 and 4 weeks prior to transport to ED	LTCF charts
	Number of times family physician attended a team meeting during study time period	LTCF charts
	Family physician visits to patient 3 months prior to most recent EHS call	LTCF charts
	Number of notes in chart from family physician during time period	LTCF charts
	Health care profession type who made onsite assessment	LTCF charts
	Any investigations (i.e. diagnostic imaging, blood work, EKG, other) in the 7 days prior to EHS call	LTCF charts
	LTC-CGA present	LTCF charts; Hospital charts
	Percentage of cases where facility was able to reach the family physician prior to EHS call	LTCF charts
	Percentage of cases with an onsite assessment by a family physician prior to EHS call	LTCF charts
	Number of times EHS (ECP and/or emergency paramedics) involved during time period	LTCF charts
	Number of patients transported to ED by ambulance	LTCF charts; EHS database
	Proportion of patients who are transported to ED who have advance comfort care directive requesting no transfer to hospital/ acute care	LTCF charts; EHS database
	Whether LTC-CGA sent with resident to ED	Hospital charts
	If ECP involved in call	EHS database
	If ECP involved, whether they consulted with EHS physician	EHS database
	If ECP involved, whether they consulted with family physician	EHS database
	Length of EHS call	EHS database
	Ambulance offload time in ED	EHS database, Hospital charts

	Length of Stay in ED	Hospital charts
	Percentage of residents who were transported to ED that were admitted to hospital	Hospital charts
	Length of stay in hospital	Hospital charts
	Percentage of transferred residents who returned to LTCF upon hospital discharge	LTCF charts ; Hospital charts
Clinical and Quality of Care	Number of assessments and treatments provided by EHS	EHS database
	Admitting diagnosis	Hospital charts
	Death rate in hospital	Hospital charts
	Influenza vaccination rates	LTCF charts
	Rates of falls	LTCF charts
	Pressure wound care	LTCF charts
	Polypharmacy rates	LTCF charts
Safety Outcomes	Relapse rate back to EHS system (number of patients seen by ECP and/or paramedics and not transported who had unexpected repeat 911 call made for them within 48 hours for a related reason)	EHS database
